# Banxia Xiexin Decoction in the treatment of chronic atrophic gastritis: A protocol for systematic review and meta-analysis

**DOI:** 10.1097/MD.0000000000022110

**Published:** 2020-10-16

**Authors:** Qing Ji, Ying Yang, Xiaolei Song, Xu Han, Wenlin Wang

**Affiliations:** a College of Nursing, School of Medicine and Health of Anyang Vocational and Technical college, Anyang; b Department of Rehabilitation Medicine, the People's Hospital of SND, Suzhou; c Department of Orthopedics, Angang Staff General Hospital, Anyang; d Department of TCM, National Medical Hall of Nanjing University of Traditional Chinese Medicine; e Department of TCM, Nanjing University of Science and Technology Hospital, Nanjing, China.

**Keywords:** Banxia Xiexin decoction, chronic atrophic gastritis, protocol, randomized controlled trials, systematic review

## Abstract

**Background:**

Chronic atrophic gastritis (CAG) is a common digestive disease. Without active treatment, it may induce gastric cancer. Western medicine has a certain effect on chronic atrophic gastritis, but there are many adverse reactions after long-term medication, and the disease is prone to relapse after treatment, which will affect the health and life of patients. Traditional Chinese medicine has obvious advantages in the treatment of chronic stomach diseases with reliable effect. A number of clinical data have also confirmed that Banxia Xiexin decoction has significant effect in the treatment of chronic atrophic gastritis, but there is no evidence of evidence-based medicine. Therefore, this study aims to explore the clinical efficacy and safety of Banxia Xiexin decoction in the treatment of chronic atrophic gastritis by means of systematic evaluation.

**Method:**

Databases including PubMed, The Cochrance Library, Embase, Web of Science, CNKI, VIP, and Wanfang were searched by computer. Besides, Baidu Scholar and Google Scholar were manually searched, and all randomized controlled trials of Banxia xiexin decoction for the treatment of chronic atrophic gastritis were collected. The retrieval time was from the establishment of the database to July 31, 2020. After 2 reviewers independently screened the literature, extracted the data and evaluated the bias risk of the included study, RevMan5.3 software (developed by the UK's International Cochrane Collaboration) was used to analyze the data.

**Results:**

In this study, the effectiveness and safety of Banxia Xiexin decoction for the treatment of chronic atrophic gastritis were evaluated by the clinical efficiency, traditional Chinese medicine syndrome score (traditional Chinese medicine syndrome score), quality of life score, gastrin level, epidermal growth factor, eradication rate of *helicobacter pylori* and incidence of adverse reactions.

**Conclusion:**

This study will provide reliable evidence for the clinical application of Banxia Xiexin decoction in the treatment of chronic atrophic gastritis.

**Ethics and dissemination:**

The private information from individuals will not be published. This systematic review also will not involve endangering participant rights. Ethical approval is not required. The results may be published in a peer-reviewed journal or disseminated in relevant conferences.

**OSF registration number:**

DOI 10.17605/OSF.IO/7K6QW

## 1 Introduction

Chronic atrophic gastritis (CAG) is a type of chronic gastritis that is a chronic stomach disease in which repeated injury to the gastric mucosa epithelium results in a diminution of mucosal glands, or with intestinal metaplasia and/or pseudopyloric metaplasia.^[1,2]^ A multicentric epidemiological survey in 2014 showed that the prevalence rate of CAG is high in China at present, about 25.8%.^[3]^ CAG is related to *H pylori* infection, environmental factors, and genetic factors, among which *H pylori* infection is the main pathogenic cause of the disease.^[4,5]^ CAG patients may have no obvious clinical symptoms, or show symptoms such as upper abdominal pain, nausea, and loss of appetite.^[6,7]^ With the progression of the disease, CAG patients may have pathological changes such as thickening of mucosal muscle layer and intestinal metaplasia,^[8]^ increasing the risk of cancer.^[9]^ Therefore, active and effective treatment can not only improve patients’ clinical symptoms and improve their quality of life, but also prevent the occurrence of cancer.^[10]^ Western medicine has a single effect on CAG, and long-term use may cause adverse reactions, which makes the treatment difficult.^[11]^

Banxia Xiexin decoction is a classic prescription in Treatise on Febrile Diseases (Shanghan Lun) of the Han Dynasty of China, which can be used for diseases such as functional dyspepsia,^[12]^ gastroesophageal reflux disease^[13]^ and colon cancer.^[14]^ The prescription has the effect of harmonizing liver and spleen, dissipating distension syndrome, and dispersing nodule, enriching qi, and nourishing Yin. Studies have shown^[15]^ that Banxia Xiexin decoction can significantly improve the clinical symptoms of patients with CAG, with fewer adverse drug reactions, so it is widely used in clinical practice.

In recent years, many clinical studies have shown that Banxia Xiexin decoction combined with Western medicine has a significant effect on the treatment of CAG. However, there are differences among clinical trials in terms of research program and efficacy, resulting in uneven research results and to some extent, affecting the promotion of Banxia Xiexin decoction. Therefore, this study collected randomized controlled trials of Banxia Xiexin decoction combined with Western medicine in the treatment of CAG, and objectively evaluated the efficacy and safety of Banxia Xiexin decoction in the treatment of CAG according to the systematic evaluation method of Cochrane Collaboration, providing reliable reference for the clinical application of Banxia Xiexin decoction in the treatment of CAG.

## 2 Methods

### 2.1 Protocol register

This protocol of systematic review and meta-analysis has been drafted under the guidance of the preferred reporting items for systematic reviews and meta-analyses (PRISMA-P). Moreover, it has been registered on open science framework (Registration number: DOI 10.17605/OSF.IO/7K6QW).

### 2.2 Ethics

Since this is a protocol with no patient recruitment and personal information collection, the approval of the ethics committee is not required.

### 2.3 Eligibility criteria

We will collected all available randomized controlled trails on Banxia xiexin decoction treatment for CAG, regardless of blinding, publication status, region, but Language will be restricted to Chinese and English.

#### 2.3.1 Research objects

Patients who meet the diagnostic criteria for CAG, regardless of their nationality, race, age, gender, and course of disease.

#### 2.3.2 Intervention measures

The control group was treated with western medicine alone, and there were no restrictions on the type, dose, and course of treatment of western medicine. The treatment group was treated with Banxia Xiexin decoction plus or minus combined with western medicine, and there were no restrictions on the dosage form, dosage, and medication methods of Banxia Xiexin decoction.

#### 2.3.3 Outcome indicators

(1) Primary outcome: the overall effective rate;
(2) secondary outcomes:
(3) traditional Chinese medicine (TCM) syndrome score;
(4) quality of life score;
(5) gastrin level;
(6) epidermal growth factor;
(7) *H pylori* eradication rate;
(8) incidence of adverse reactions.

### 2.4 Exclusion criteria

(1) Repeatedly published studies;
(2) the study that published as abstract or study whose data is incomplete, and relevant literature cannot be obtained after contacting the author;
(3) studies with obvious data errors;
(4) studies with the treatment group was the Banxia Xiexin decoction alone or combined with other TCM therapies, such as combined with other TCM compounds, acupuncture, moxibustion, acupoint sticking, etc;
(5) literature with high bias risk assessed by randomization or allocation concealment.^[16]^

### 2.5 Retrieval strategy

CNKI, Wanfang Data, VIP, PubMed, Cochrane Library, Embase, Web of Science, and other databases were searched by computer, and the retrieval time was from the date of establishment to July 31, 2020. The Chinese keywords of the search were “chronic gastritis(man xing wei yan)”, “CAG(man xing wei suo xing wei yan)”, “Banxia Xiexin decoction(ban xia xie xin tang)”, etc. The English keywords of the search were “gastphic,atrophic” “atrophic Gastritides,” “atrophic gastritis” and “Banxia Xiexin decoction”. In both Chinese and English, subject words were combined with free words. In addition, manual search was conducted on Baidu academic and Google academic, and all randomized controlled trials of Banxia Xiexin decoction for the treatment of CAG were collected. The search strategy (PubMed) is shown in Table 1.

**Table 1:**
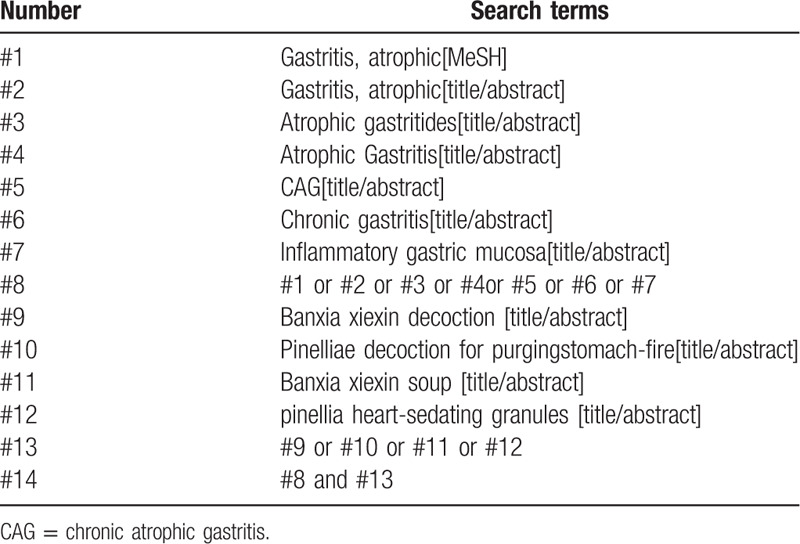
Search strategy in PubMed database.

### 2.6 Data screening and extraction

**Figure 1:**
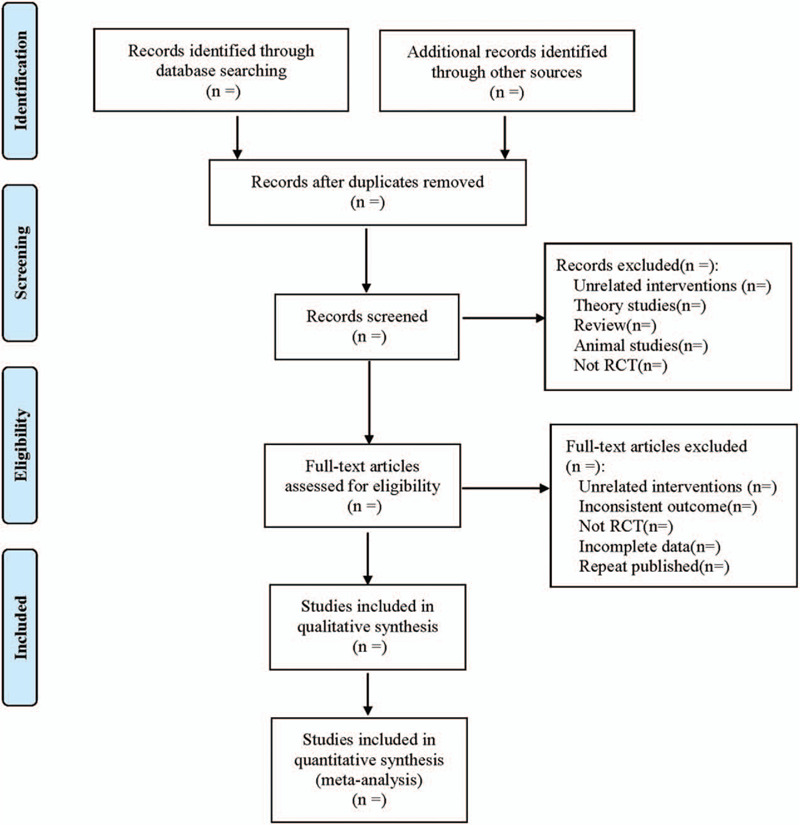
Flow diagram.

Cochrane Handbook for Systematic Reviews of Interventions Printable version 5.0 is developed by the Cochrane Collaboration International was used as a reference for the method of selection in the study, according to the preferred reporting items for systematic reviews and meta-analyses flow chart, Endnote X7 (developed by The National Institute of Scientific Information) document management software was utilized by 2 researchers to independently screen the documents based on the above inclusion and exclusion criteria, duplicate references were removed from each database, and clearly unqualified studies were excluded based on title and abstract, and the remaining studies were further screened based on the full text. During the screening, the 2 researchers read the literature independently for screening, and then the 2 researchers checked the results of the included trial with each other, and discussed with the third researcher to determine whether or not to include studies in which there was disagreement. At the same time, Excel 2013 was used to extract relevant information, including:

(1) Clinical research (title, first author, publication year and month, sample size, sex ratio, average age, average course of disease);
(2) Intervention measures (name, dosage, course of treatment of western medicine used in the control group; the dosage form, dosage, and course of treatment of Banxia Xiexin decoction or usage and dosage of other western medicine used in the treatment group);
(3) Evaluation factors of risk bias in randomized controlled studies;
(4) Observation indicators. The literature screening process is shown in Figure 1.

### 2.7 Literature quality evaluation

The risk of bias for each eligible study will be assessed by 2 researchers respectively according to the Cochrane Collaboration's tool including seven terms. According to these criteria (random sequence generation, allocation concealment, blinding, incomplete data, selective result reports, and other bias), risk of bias is classified into the following levels: unclear, low, and high risk of bias. Any divergences will be solved through discussion by a third researcher.

### 2.8 Statistical analysis

#### 2.8.1 Data analysis and processing

The RevMan 5.3 software (developed by the UK's International Cochrane Collaboration) was used for statistical analysis. For dichotomous variables, relative risk was used for statistics. For continuous outcomes, weighted mean difference was selected when the tools and units of measurement indicators are the same, standardized mean difference was selected when the tools and units of measurement indicators are different, and all the above were represented by effect value and 95% confidence interval. The heterogeneity was determined by χ^2^ and I^2^ values. if (*P*≥. 1, I^2^≤50%) indicated low heterogeneity, fixed effect model was used for Meta analysis. If(*P*<. 1, I^2^>50%)indicated heterogeneity among studies, and the source of heterogeneity would be explored through subgroup analysis. If there was no obvious clinical or methodological heterogeneity, it would be considered as statistical heterogeneity, and the random-effect model would be used for analysis. Descriptive analysis was used instead of Meta analysis if there was significant clinical heterogeneity between the 2 groups and subgroup analysis was not available.

#### 2.8.2 Dealing with missing data

If there is missing data in the article, contact the author via email for additional information. If the author cannot be contacted, or the author has lost relevant data, descriptive analysis will be conducted instead of Meta analysis.

#### 2.8.3 Subgroup analysis

Subgroup analysis was conducted according to the different western drugs used in the intervention group. Subgroup analysis was carried out according to different dosage forms of Banxia Xiexin. Subgroup analysis was conducted according to the course of treatment of Chinese herbal compound.

#### 2.8.4 Sensitivity analysis

In order to determine the stability of outcome indicators, sensitivity analysis was used to analyze each outcome indicator.

#### 2.8.5 Assessment of reporting biases

Funnel plots were used to assess publication bias if no fewer than 10 studies were included in an outcome measure. Moreover, Egger and Begg test were used for the evaluation of potential publication bias.

#### 2.8.6 Evidence quality evaluation

The Grading of Recommendations Assessment, Development, and Evaluation will be used to assess the quality of evidence. It contains 5 domains (bias risk, consistency, directness, precision, and publication bias). And the quality of evidence will be rated as high, moderate, low, and very low.

## 3 Discussion

The prevalence of CAG increases with the increase of age,^[1]^ but there is no significant correlation with gender.^[17]^ Clinically, the treatment of CAG focuses on the removal of etiology, relief of symptoms and improvement of gastric mucosal histology,^[18]^ including regular diet, symptomatic treatment and *H pylori* eradication treatment. In the treatment of CAG patients by modern medicine, symptoms of some patients are difficult to improve, recurrent attacks, and other problems, while TCM has obvious advantages in improving symptoms, and also plays a role in protecting the gastric mucosa of patients and delaying the development of the disease.

In Chinese medicine, CAG is classified as “distension syndrome(Pi Man)”, “stomachache(Wei Wan Tong)” and other diseases. Six pathogenic factors, emotional disorders, and internal injuries of diet can all damage the spleen and stomach, causing dysfunction of spleen and stomach elevation, then further causing spleen deficiency and dampness, dampness, and turbidity stasis blocking stomach collaterals, the loss of supply of nutrients of stomach, and gastric mucosa atrophy. Therefore, weakness of spleen and stomach and imbalance of qi are the main pathogenesis of this disease.^[19]^ Banxia Xiexin decoction consists of Banxia (*Rhizoma Pinelliae*), Huangqin (*Radix Scutellariae*), Ganjiang (*Rhizoma Zingiberis*), Renshen (*Radix Ginseng*), Zhigancao (*Radix Glycyrrhizae Uralensis*), Huanglian (*Rhizoma Coptidis*), Dazao (*Fructus Jujubae*). Banxia (*Rhizoma Pinelliae*) is the monarch medicine, which has the effect of dissipating distension syndrome and dispersing nodule, drying dampness, and reducing phlegm. It can be combined with Renshen (*Radix Ginseng*) to strengthen the spleen and stomach, and effectively improve the clinical symptoms of patients.^[20]^ Huangqin (*Radix Scutellariae*) and Huanglian (*Rhizoma Coptidis*) can clear heat and detoxify, and have a certain inhibitory effect on *H pylori*.^[21]^ Ganjiang (*Rhizoma Zingiberis*) warm the middle-jiao and disperse cold; Zhigancao (Radix Glycyrrhizae Uralensis) and Dazao (Fructus Jujubae) mixed with all the medicine, the whole prescription plays a role in invigorating the spleen and invigorating the qi, to be acrid to diffuse and bitter to descend. Modern pharmacological studies have shown that Banxia xiexin Decoction can protect gastric mucosa by inhibiting *H pylori* and lowering the level of TNF-α in serum.^[22]^ Banxia Xiexin decoction can inhibit the activation of NF-κB/STAT3 signaling pathway and reduce the expression of inflammatory cytokines TNF-α, IL-6, IL-8 and proto-oncogene BCL-2, thereby inhibiting the occurrence and development of precancerous lesions.^[23]^ Clinical studies also showed^[24]^ that compared with the control group, the total effective rate of Banxia Xiexin decoction combined with Western medicine was higher, the degree of gastric mucosal inflammation disappeared significantly, and the clinical symptoms were also significantly improved.

Through this study, the efficacy and safety of Banxia Xiexin decoction in the treatment of rheumatoid arthritis can be systematically evaluated, and the difference in efficacy between banxia Xiexin decoction plus or minus combined with western medicine and western medicine alone can be obtained. In addition, the adverse reactions of Banxia Xiexin decoction in clinical practice can be learned, which is conducive to the promotion of clinical use. However, this study also has some limitations, including few literatures and small sample size. In addition, because the treatment group was treated with the addition and reduction of Banxia Xiexin decoction, rather than the original prescription of Banxia Xiexin decoction, the medication and dosage were different, and there was a certain clinical heterogeneity. In addition, this study only searched English and Chinese literature, and may ignore studies or reports in other languages, with certain publication bias. Therefore, high-quality and large sample literature support is still needed, so as to improve the reliable basis for clinicians and patients to use Banxia Xiexin decoction to treat CAG.

## Author contributions

**Data collection:** Qing Ji and Ying Yang.

**Funding support:** Wenlin Wang.

**Literature retrieval:** Xiaolei Song and Xu Han.

**Software operating:** Ying Yang.

**Supervision:** Wenlin Wang.

**Writing – original draft:** Qing Ji and Ying Yang.

**Writing – review & editing:** Qing Ji and Wenlin Wang.
